# The trail making test in India

**DOI:** 10.4103/0019-5545.33258

**Published:** 2007

**Authors:** Triptish Bhatia, Vandana Shriharsh, Saurabh Adlakha, Vivek Bisht, Kapila Garg, Smita N. Deshpande

**Affiliations:** Department of Psychiatry, Dr. Ram Manohar Lohia Hospital, New Delhi - 110 001, India

**Keywords:** Cognitive dysfunction, schizophrenia, trail making test

## Abstract

The trail making test (TMT) is a short and convenient estimate of cognitive functions, principally attention and working memory. Like most neuropsychological tests, it is derived from and primarily applicable to English-speaking individuals. Norms for other ethnic minorities may differ significantly. The application of majority or mixed norms to specific ethnic subcultures may introduce systematic bias. To examine the impact of an English test on primarily nonEnglish-speaking individuals, outpatients attending the dermatology department of a large Indian hospital (*n* = 120) were asked to complete the English version of the TMT. The time taken to complete the TRAILS was unexpectedly long, although all the subjects scored within normal limits on the modified mini mental status examination and a test for general knowledge. Possible reasons for the delayed completion times are discussed below.

The trail making test (TMT) is a brief paper and pencil neuropsychological test often used for screening for cognitive impairment.[1] The TMT was a standard component of the Halstead-Reitan Battery[2] which measures cognitive dysfunction. Some authors[2] interpret the TMT, specifically Part B, as an executive task. Part A appears to be dependent primarily on the efficiency of visual scanning and psychomotor speed. In contrast, Part B consists of circles, some of which contain numbers and others letters. The alternation between serial sequences of letters and numbers is thought to require executive control, specifically, flexibility of thinking and greater demand for working memory. Relatively few studies have evaluated the TMT using languages other than English. Stanczak *et al.*[3] reported that healthy Sudanese control individuals performed worse than US controls. Their scores were similar to those of US brain-damaged individuals even after controlling for age and education and using the Sudanese and English versions in the Sudanese and US populations respectively.

Such factors could well lead to diagnostic errors while evaluating cognitively impaired individuals in different cultural contexts. Other ethnic groups have also been investigated in the USA. Using only Part A of the TMT, older Japanese-American individuals (≥ 65 years of age) showed significant age effects on TMT scores[4] (mean score 79.8 ± 49.2 seconds). Among Italians, TMT scores were found to be correlated with age, education and general intelligence, women taking longer than men to complete the Trails A.[5] In spite of being simple, inexpensive and easy to administer, the TMT has not received widespread acceptance in India even though English is widely spoken and taught in this country. This is a preliminary study of the trail making test in a subset of normal Indian population.

## MATERIALS AND METHODS

This study was conducted at the outpatient service of the Department of Dermatology at a publicly funded tertiary care hospital in New Delhi. Funded by the Government of India, this hospital has 800 inpatient beds and caters to more than 2,00,000 outpatients annually.

Every fifth outpatient was included in the study population. Only persons who knew the English alphabet and were above fifteen years of age were included in the study. All patients thus recruited were screened for psychiatric or neurological illnesses. Individuals with such problems were excluded from the study. About 160 patients were screened and out of these 120 agreed to participate. No individual identifiers were recorded and the data generated was thus anonymous. All participants provided informed verbal consent in accordance with the regulations of the Ethics Committee of the hospital.

### Data obtained

1. Demographic:
  The age, gender, educational and occupational status of subjects and occupation of the heads of their households were recorded using the demographic data sheet of the Diagnostic Interview for Genetic Studies.[6] Subjects with any kind of psychiatric history or symptoms were not included. Detailed psychiatric evaluations were not performed.
2. TMT A and B
  The TMT consists of two parts, A and B. Part A consists of one sample test and one task. The numbers are randomly printed on the sample worksheet. The subject is required to join consecutive numbers in order by drawing connecting lines. The worksheet consists of numbers 1 to 25. The time taken to join consecutive numbers is taken as the subject's score.
  Part B consists of a sample test as well as the main task. The numbers 1 to 4 and the letters A to D are presented on a sample worksheet. The numbers 1 to 13 and letters A to L are presented on the task worksheet. The participant is required to alternate between numbers and letters as s/he proceeds in an ascending sequence. Subjects are asked to connect numbers-alphabets as fast as they can. The examiner points out errors as they occur so that the subject can complete the test without errors. The score is only based on the time taken.
  If the time taken to complete Part A is less than the time taken to complete Part B, the subject is considered to have difficulties in complex conceptual tracking. In general, performance is considered to be impaired if scores exceed 40 seconds for part A and 91 seconds for part B.[7]
  In the present study, the time taken to complete the test was recorded. If an error occurred, the participant was directed to correct it but the clock used to time the test was not stopped. Total time taken to complete the task was considered as the final score. Number of errors was not recorded.
3. Modified mini mental status exam (MMMSE)
  The MMMSE consists of 35 items (each with a score of 1) that assess global cognitive functioning across the following domains: Orientation, registration, attention and calculation, memory recall, language and cognitive state.[8] A person with a total score ≤ 15 is considered cognitively impaired.
4. Information subtest of verbal adult intelligence scale of the Post Graduate Institute Battery of Brain Dysfunction (PGIBBD)

The PGIBBD has been developed and tested in India, used to evaluate cognitive function following brain damage. Only the Hindi version of the information subtest out of four subtests consisting of 33 items was used in the present study as a test of general and fixed knowledge acquired by the subject. The subject's response to the questions was recorded verbatim. The test was discontinued if seven consecutive failures were recorded. One point was given for each correct response.

Two authors (SA and VB) administered the tests. They were trained and supervised by two other co-authors (TB and SND).

### Statistical analysis

Linear regression was carried out to examine the effect of different variables on the time taken to complete Task A, Task B, Tasks A and B combined and executive functioning. The variables considered were age, sex, education, marital status, MMMSE and information score of the PGIBBD test. Version 10.5 of SPSS was the statistical package used.

## RESULTS

Data was generated for 120 participants: 34 women and 86 men. Only six participants declined to participate due to lack of time and inadequate knowledge of English. The mean age of the sample was 30.13 ± 13.06 years and the mean education was 12.13 school years [Table 1].

**Table 1:** Comparison of time taken for trail making test in two groups

Variable	Male (n = 86)	Female (n = 34)	Total sample (n = 120)
Age	29.9 (12.5)	30.62 (13.78)	30.11 (12.83)
Duration of education (years)	12.06 (3.1)	12.29 (3.16)	12.13 (3.13)
Information score	21.16 (5.47)	20.1 (5.13)	20.89 (5.38)
MMSE score	32.66 (2.31)	31.79 (2.48)	32.41 (2.38)
Mean time for task A	75.13 (30.81)	76.03 (34.69)	75.38 (31.81)
Mean time for task B	146.39 (49.7)	161.56 (49.25)	150.69 (49.84)
Total time taken (Tasks A+B)	221.52 (67.45)	237.59 (70.34)	226.07 (68.37)
Executive function Time B-A	71.27 (47.86)	85.53 (48.07)	75.3 (48.15)

Values are reported as means (standard deviations in brackets).

All subjects scored within normal limits on the MMMSE (mean score 32.41) and the PGIBBD information subtest (mean score 20.89, *i.e*., 60% correct answers).

The total mean time taken to complete both tasks of the TMT was 226.07 ± 68.37 seconds [Table 1]. The mean time taken on task A of the TMT was 75.3 seconds and for Task B was 150.69 ± 49.84 seconds. The mean time taken to complete Task A for males was 75.13 seconds and for females, 76.03 seconds

The results of regression analysis [Table 2] revealed that the number of years of education was the most significant predictor variable. Age affected the trail making tests when Trail A and Trail B were taken together suggesting younger people scored better than older people.

**Table 2:** Predictors of trail making test in the sample

(Regression analysis)	

Dependent variables	Predictors
Task A	Education
	B = −3.089 (P<0.001)*
Task B	Education
	B = −5.278, (P<0.001)*
Task A+B	Age
	B = −1.29 (*P* = 0.043)*
	Education
	B = −53.070 (*P*<0.001)*
Task B-A	Education
	B = −2.426 (*P* = 0.048)*

* All these values are statistically significant. B = Regression coefficient

## DISCUSSION

We compared performance on the TMT among Indian participants, whose mother tongue was not English and who did not have a history of psychiatric treatment. Education was the most important predictor of performance on both tasks of the TMT taken together, taken separately as well as on executive functioning (Task B-A). Education might also have facilitated easy performance on a novel test. The level of education among participants was inversely related to the time for completion of tests in other published studies.[7,9]

Our participants took much more time-75.38 ± 31.81seconds for Task A and 150.69 ± 49.84 seconds for Task B (mean age 30.11 years and mean education 12.13 years) than the accepted cutoff value of TMT, which is considered to be 40 seconds for Task A and 91 seconds for Task B.[10,11] However in Heaton's[12] study, mean completion time reported was 79.8±42.5 seconds for Trail B (mean age of 34.6±12.83 years and mean education 12-13 years).[13] A larger study with a bigger sample is needed to replicate cut offs for this test in the Indian population so that these cut offs can be generalized.

Although the participants in our study knew the English alphabet and numbers; they did not use English regularly. We considered translating numbers and letters into Hindi, but due to difficulties with matching the letters used in the TMT, this was considered not feasible. Lack of familiarity with cognitive tests in certain cultures may lead to scores that differ from American individuals.[3] These types of cognitive tasks are also not regularly used in our culture. Differences in cognitive styles, influenced by socio-cultural factors, may also influence performance on some tests. Stanczak *et al.*[3] argued that when a test such as the TMT is adapted for cross-cultural use, modifications of the original test stimuli could alter the instrument's construct validity.

In summary, scores on the Trails A and B tests in a relatively small subpopulation of Indian adults are presented. These results differ from published results from other cultural groups. Variations in the educational level as well as cultural variables may explain the differences.

The main limitation of the study is that it is not able to suggest an alternative for the population who do not know English.
